# Semiconductor-Based Photocatalytic, Photoelectrochemical, and Photovoltaic Solar-Energy Conversion

**DOI:** 10.1155/2014/695204

**Published:** 2014-06-12

**Authors:** Dengwei Jing, Jinwen Shi, Patrick Meyrueis, Han Zhou

**Affiliations:** ^1^International Research Center for Renewable Energy, Xi'an Jiaotong University, Xi'an 710049, China; ^2^Photonics Systems Laboratory, University of Strasbourg, 67412 Illkirch, France; ^3^Department of Colloid Chemistry, Max Planck Institute of Colloids and Interfaces, Research Campus Golm, 14476 Potsdam, Germany

Semiconductor-based photocatalytic, photoelectrochemical, and photovoltaic solar-energy conversion has been considered very promising to address the energy and environmental challenges. At the heart of the technology is the lack of suitable semiconductor materials and efficient systems that can perform the solar-energy conversion efficiently and inexpensively. Much work needs to be done in this research field, for instance, full control of growth characteristics and physicochemical properties of targeted semiconductor materials and rational design of systems efficiently utilizing incident solar light.

The main objective of this special issue is to bring together researchers and engineers working on semiconductor-based solar-energy conversion and to bridge between fundamental materials research and solar-energy conversion. After critical peer review, 6 papers of high quality were selected from many submissions. The topics of all the accepted papers are closely related to the subject of this thematic issue. As per photovoltaic solar-energy conversion, N. H. Samrat et al. reported the modeling, control, and simulation of a PV-wave energy hybrid power generation system that is assumed to be used for island electrification in Malaysia. The concept is interesting and theoretically applicable. J. Zheng et al. described an interesting solar tracking error analysis for Fresnel reflector. Considering the rapidly increased use of Fresnel reflector in concentrating PV, the error analysis could be of importance for the design of such PV system. Tseng et al. reported an interleaved boost converter with coupled inductor for PV energy conversion. The proposed soft-switching boost converter uses an interleaved method to increase its power density and coupled-inductor technology to extend its step-up voltage ratio. The proposed strategy has been demonstrated to be quite suitable for PV energy conversion.

Other three papers are related to the photocatalytic, photoelectrochemical application of semiconductor film as electrodes or powders as photocatalysts. Their work represented important progress in the design of semiconductors with multiple compositions, functional nanostructures, and, most importantly, enhanced properties. So the reported work is expected to be of interest for a broad range of readership.

In summary, semiconductor-based photocatalytic, photoelectrochemical, and photovoltaic solar-energy conversion is gaining more and more attention, and the papers presented in this special issue represent the important progress in their research. Nevertheless, many problems remain unresolved, and more research is necessary. We look forward to new progress on the basis of and beyond the work reported in this issue.

*Dengwei Jing* *Dengwei Jing* *Jinwen Shi* *Jinwen Shi* *Patrick Meyrueis* *Patrick Meyrueis* *Han Zhou* *Han Zhou*

